# Intraspecific morphological variation of the sperm storing organ in two hermaphroditic land snail species

**DOI:** 10.1186/s40709-019-0093-y

**Published:** 2019-01-30

**Authors:** Alexandra Staikou, Evripidis Koemtzopoulos

**Affiliations:** Department of Zoology, School of Biology, Aristotle University of Thessaloniki, 541 24 Thessaloniki, Greece

**Keywords:** Spermatheca structure, Sperm storage capacity, Sperm competition, Gastropods, Environmental adaptation

## Abstract

**Background:**

Postcopulatory sexual selection is very important in species with reproductive strategies that involve multiple mating and prolonged sperm storage. The sperm storage organ has been hypothesized to evolve in response to different levels of sperm competition in several species while population density has been considered as a factor that approximates sperm competition risk and intensity in the field. Apart from population density, local microclimatic conditions may also play a role in determining sperm competition levels in natural populations of land snails by affecting their chances of encountering mates. The goal of this study was to investigate the variation of the structure of the sperm storage organ in the simultaneously hermaphroditic land snails *Helix lucorum* and *Cepaea vindobonensis* occurring sympatrically in two sites which differed in habitat humidity. The populations of both species from the two sites, also differed in density and in duration of reproductive period. Multiple samples were taken from each population in order to test for temporal variation.

**Results:**

In both species, the spermatheca consisted of a simple fertilization chamber and a variable number of lateral tubules. The length of the spermatheca showed no temporal or spatial differentiation nor did it show any correlation with snail size. The number of tubules in *Helix lucorum* ranged from five to sixteen and in *Cepaea vindobonensis* from one to eight and in both species a significant difference of this trait between the two study sites was detected. In *Cepaea vindobonensis*, the difference in tubule number led to difference of the total tubule length which reflects sperm storage capacity of the spermatheca but this was not the case with *H. lucorum* in which no increase in total tubule length was detected.

**Conclusions:**

Intraspecific variation in the spermatheca was observed in both snail species studied. The variation observed was independent of snail size, and reproduction status, while the two species responded differently to higher sperm competition levels.

## Background

Sperm storage is a common phenomenon in most animals with internal fertilization and, as it temporarily separates copulation from fertilization, it can be adaptive in ecological diverse habitats [1]. In this context sperm storage may affect life history and mating system and additionally provides increased opportunity for post-copulatory sexual selection, including sperm competition, cryptic female choice or sexual conflict [1, 2].

Females of most sperm storing animals, such as birds [2, 3], reptiles [e.g. 4] amphibians [5], insects [6, 7] and gastropods [8–11], possess highly specialized structures in their genital systems where sperm is kept alive and capable of fertilization for a variable time period. This diversity in sperm storing structures may reflect functional aspects as for instance differential storage capacity demands, which may arise due to divergence in life history traits, e.g. longevity, egg productivity, sperm utilization efficiency, or selection for functional design in order to match sperm morphology [12]. Females of iteroparous species, which mate and lay eggs multiple times during their lifetime, may require more complex organs than females of semelparous species to provide space and nutrients to sperm stored in order to keep it viable [13, 14]. Additionally, female reproductive morphology may vary in different habitats, because of adaptations of life-history traits, e.g. longevity to local conditions [14, 15].

Moreover, theoretical and empirical approaches suggest that the complexity of the sperm storing organ in several animal taxa might have evolved in response to post-copulatory sexual selection pressures [16]. This applies to taxa with multiple mating and delayed fertilization in relation to insemination, a condition that results in overlapping ejaculates from multiple donors being stored and compete for fertilization (sperm competition) [17]. In several taxa the females may also evolve adaptations that favor selective use of sperm from certain donors over others (cryptic female choice) [18].

The hermaphroditic pulmonate snails and slugs (Gastropoda: Pulmonata) show diverse and very complex reproductive organs and mating behaviours. During the last years several studies have tried to explain the evolution of such complexity in pulmonates [14, 19–22]. A characteristic of their complex reproductive anatomy is the presence of sperm digesting and sperm storing organs, as well as the presence of the love dart (in which snails shoot to their mating partner during courtship) and associated mucus glands. Furthermore, multiple mating and delayed fertilization suggest that post copulatory sexual selection processes (sperm competition and/or cryptic female choice) may function in these animals.

Despite the fact that a good number of studies refer to morphological variation of the dart apparatus or the sperm storing organ [23], the adaptive significance of such variation has attracted little attention with only a handful of studies being conducted on a very limited number of species [9, 24–26]. Two empirical studies failed to establish a relationship between the degree of complexity of the sperm storing organ and sperm competition intensity as assessed either by population density [9] in *Arianta arbustorum*, or by direct observations of mating frequency in laboratory experiments in *Cornu aspersum* [10]. Nevertheless, a recent study on *Helix aperta* populations revealed a strong correlation of spermatheca storage capacity to population density [27].

According to Beese et al. [14], several factors have been suggested to affect the evolution of complex spermathecae and darts in many stylommatophoran families like body size, reproductive strategy and habitat type. Habitat type characteristics have a direct influence on populations as they affect growth and reproduction and, most importantly, they also influence populations indirectly through phenotypic plasticity of traits determining interactions between individuals [28]. The influence of habitat characteristics on selection pressures posed on animals through differentiation of their mating activity has been very little studied in stylommatophoran species. Relevant studies until now have focused on variation among species while intraspecific variation has largely been ignored [14]. Within the same species, mating systems may also vary because of differences in population density and local environmental conditions [14]. For instance, in habitats with differing humidity regimes, land snail species may differentiate reproduction timing and duration [29]. Such differences in populations of species with wide distribution ranges may lead in differential intensity of sexual selection produced by differential opportunities to seek and assure mating partners.

*Helix lucorum* Linnaeus, 1758 and *Cepaea vindobonensis* (Férussac, 1821) are two snail species frequently found in habitats of differing climatic zones in North Greece where they exhibit intraspecific differences of their activity and reproduction cycles. Specifically, snail populations in Mediterranean type habitats respond to the dry summer period (June–August) by entering aestivation and restrict their reproductive period either in spring months (March–May) [30] or in autumn [29]. Snail populations in humid inland habitats lack a summer aestivation period and may extend their reproductive period in summer months [31, 32]. Such intraspecific differences in timing and duration of the reproductive period may well result in differences in mating opportunities of the snails during each reproductive period. This, in turn, possibly affects the number of matings achieved and hence the levels of sperm competition intensity faced by snails of the different populations.

The aim of the present study was (a) to study the morphology of the fertilization pouch-spermatheca complex (FPSC) in two populations of *H. lucorum* and *C. vindobonensis* of differing activity and reproduction characteristics and (b) to examine if variation in reproductive activity of populations studied, is reflected into morphological variation of the sperm storing organ.

## Methods

### Study sites and phenology of the studied species

The life cycle and population dynamics of the two helicoids, *H. lucorum* and *C. vindobonensis*, have been studied several years ago in the Logos region of Edessa (N40°47′42″, E22°3′33″) [31, 32]. The climate of the region is of the humid Mediterranean type and the study area is situated under the local waterfalls. The wet season lasts from September to May while even the summer months (June–August) are characterized by frequent rainfalls and elevated humidity. Both snail species hibernate during winter but they do not aestivate during summer. Breeding season for both species starts in the middle of April and is extended to mid-June for *C. vindobonensis* and to late August for *H. lucorum*. Multiple mating has been observed in *H. lucorum* during the study of its life cycle and population dynamics (A. Staikou, unpublished data).

The same snail species occur sympatrically in another habitat in the coastal area of the central part of Macedonia in North Greece near the river Axios (N40°42′17″, E22°40′39″). The climate of the latter region is of the semi-arid Mediterranean type with dry summers which cause snails to aestivate. Breeding season for both species lasts from late March to early June and by the end of June the snails are found aestivating buried in the ground with their apertures sealed with one or two epiphragms [30].

### Sampling

Population density based on adult number was measured in both study sites by the method of quadrat sampling based on as series of monthly samples obtained during a whole activity period (March–November). The sampling procedure, number of samples obtained each month and sampling error were determined by the methods described in detail in previous studies [31, 32].

In an earlier study of spermatheca structure and function of the same species [33], a different pattern of temporal and spatial variation of spermatheca structures for the two species had been revealed. So, in this study for *C. vindobonensis* from both study sites we obtained samples before the beginning (April) and after the end (June) of the reproductive period referred hereafter as Edessa-April (EA), Edessa-June (EJ), Axios-April (AA) and Axios-June (AJ) samples. For *H. lucorum* we obtained different samples from the two study sites. From Axios site we obtained two samples, one in April (before) and one in June (after the reproductive period), referred hereafter as Axios-April (AA) and Axios-June (AJ) samples. From Edessa, where reproductive period is extended until the beginning of autumn, we obtained three samples, one before the beginning (April) one in the middle (June) and one after the end (September) of the reproductive period, referred hereafter as Edessa-April (EA), Edessa-June (EJ) and Edessa-September (ES) samples. These samples were obtained in order to test for temporal differentiation due to fertilization or oviposition events on the soft tissue of spermathecae (e.g. length, width) as they have been reported to be expandable [25].

### Histology

Adult snails of unknown mating history were collected from each habitat, individually wrapped in paper, packed in cardboard boxes and transferred to the laboratory. In the lab, shell diameter (D) and height (H) of each snail were measured to the nearest 0.5 mm using vernier calipers. Snails were anaesthetized using clove tree oil and water (20 drops *Eugenia caryophyllus* oil in 50 ml water) [34] and kept in this emulsion for 2 h at room temperature in order to relax and extend their body, before dissection. Finally, they were transferred and fixed in 70% ethanol.

After shell removal, the FPSCs (fertilization pouch-spermatheca complexes) were dissected and kept in 70% ethanol. Subsequently, the FPSCs were embedded in paraffin, serially sectioned at 10 μm and stained with Nuclear Fast Red and Lightgreen-Orange G [9].

The structure of each spermatheca was examined by counting the number of spermathecal tubules and by recording their branching pattern. The length of the fertilization chamber and tubules was approximated by counting the number of cross sections, starting with that section in which a certain structure was clearly separated from the structure from which it branched off. The minimum length of tubules was fixed to 50 μm (present in at least five sections). The tubule, which first branched off the fertilization chamber, was considered as the ‘main tubule’ while all other tubules, which branched off from the ‘main tubule’ or from other tubules, were called ‘lateral tubules’. For each FPSC, we counted the number of spermathecal tubules (Ntub), and calculated the length of the fertilization chamber (FCh), the length of the main tubule (MT), and the cumulative length of lateral tubules (LATt). Finally, the cumulative length of all tubules (TOTt = MT + LATt), representing sperm storing capacity of the spermatheca, was calculated.

### Statistics

For each trait within each species normal distribution and homogeneity of variances was checked and if needed a log-transformation was applied to the data to reach normality. Within species, differences among samples were tested under a General Linear Model using Nested Analysis of Variance with monthly samples and study sites as fixed factors, monthly samples nested within study sites and shell size (D, H) as covariate. Product-moment Pearson correlations were used to examine possible relationships between (a) shell size (D and H) and spermathecal traits and (b) lengths of the various spermathecal structures. All analyses were performed with SPSS ver. 23.0 (Chicago, IL, USA) provided by Aristotle University of Thessaloniki.

## Results

### Population density

Mean population density of *H. lucorum* was: in Edessa 7.40 ± 1.27 snails m^−2^ and in Axios 5.54 ± 1.44 snails m^−2^. For *C. vindobonensis* mean population density in Edessa was 2.85 ± 0.43 snails m^−2^ and in Axios 3.29 ± 0.65 snails m^−2^ (Table 1).

**Table 1:** Shell diameter (D), shell height (H) (mean ± SD), population density (adults m^−2^), and timing characteristics of the reproductive period (R.P.) of *Helix lucorum* and *Cepaea vindobonensis* from the two study sites, Edessa and Axios. Number of individuals is given in parenthesis

	*Helix lucorum*		*Cepaea vindobonensis*	
	Edessa (93)	Axios (63)	Edessa (60)	Axios (60)
Shell diameter (D)	37.88 ± 1.85^a^	44.80 ± 2.10^b^	23.98 ± 0.98^c^	24.00 ± 0.96^c^
Shell height (H)	33.32 ± 1.91^a^	40.72 ± 2.98^b^	20.20 ± 1.08^c^	20.50 ± 0.93^c^
Population density	7.40 ± 1.27	5.54 ± 1.44	2.85 ± 0.43	3.29 ± 0.65
Beginning of R.P.	Mid-April	Late March	Mid-April	Late March
End of R.P.	August	Early June	Mid-June	Early June

Different superscript letters within species indicate significant differences at *p *< 0.05

### Spermatheca morphology

The morphology of the spermatheca was studied in 276 snails (156 *H. lucorum* and 120 *C. vindobonensis*).

#### Helix lucorum

**Fig. 1:**
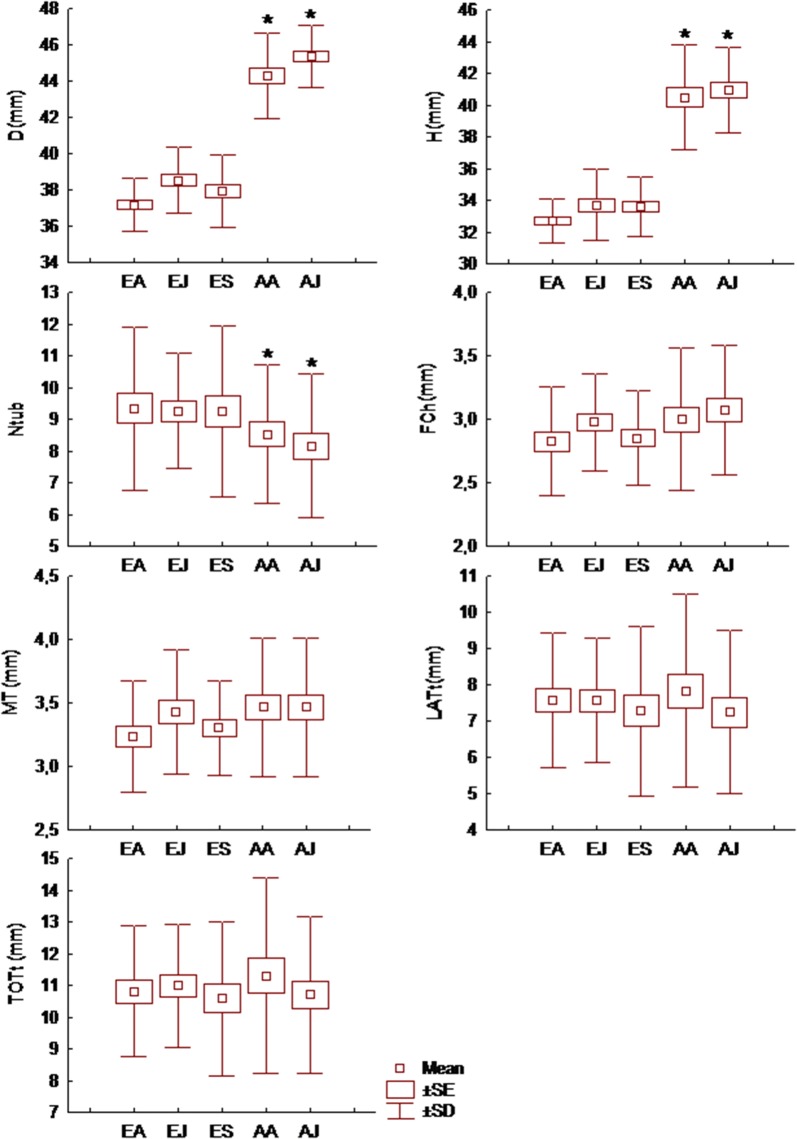
Comparison of shell size and morphological characteristics of the FPSC of *Helix lucorum* between samples taken from the two study sites. D: shell diameter, H: shell height, Ntub: number of spermathecal tubules, F.Ch.: length of the fertilization chamber, M.T.: length of the main tubule, LAT.t: the cumulative length of lateral tubules, TOT.t: the cumulative length of all tubules. EA: Edessa-April, EJ: Edessa-June, ES: Edessa –September, AA: Axios-April, AJ: Axios-June. Asterisks indicate significant differences at *p* < 0.05

Shell size, both D and H, differed significantly between the two study sites (D: t = − 21.42, *df* = 154, *p *< 0.01, H: t = − 19.25, *df* = 154, *p* < 0.01) as snails from Axios were much larger than snails from Edessa (Table 1, Fig. 1).

The FPSC, situated in the proximal genital system, consisted of the blind-ending fertilization chamber together with the spermatheca, and parts of the hermaphrodite duct and the spermoviduct. The spermatheca consisted of several tubules. The ‘main tubule” was the longest one and in the vast majority of the snails examined it was longer than the fertilization chamber except for two snails from Axios. Towards the distal end of the spermatheca, and after the ending of the fertilization chamber the main tubule became wider and in many individuals formed lateral folds. Lateral folds were also formed by lateral tubules in 50 FPSC examined. In nine (9) FPSC we observed that, after the formation of the “main tubule”, another tubule branched off directly from the fertilization chamber.

Overall, the nested analysis of variance did not reveal significant differences of the FPSC traits measured between monthly samples in the two study sites. The number of spermathecal tubules (Ntub) ranged from 5 to 16 and were significantly fewer in Axios than in Edessa (Fig. 1) (F = 6.70, *p *= 0.01). No significant correlation was found between the number of tubules and diameter of the shell (D) while a significant negative correlation was found between the number of tubules and shell height (H). The number of tubules was also significantly correlated with the cumulative length of lateral tubules (LATt) and the cumulative length of all tubules (TOTt) (Table 2).

**Table 2:** Coefficients of correlation between shell size traits and spermathecal traits of *Helix lucorum*

	D	H	Ntub	F.Ch	M.T.	LAT.t	TOT.t
D	1.00						
H	0.90*	1.00					
Ntub	− 0.10	− 0.22*	1.00				
F.Ch	0.14	0.05	− 0.05	1.00			
M.T.	0.15	0.04	0.01	0.91*	1.00		
LAT.t	0.04	− 0.12	0.59*	0.36*	0.36*	1.00	
TOT.t	0.07	− 0.10	0.54*	0.53*	0.55*	0.98*	1.00

D: shell diameter, H: shell height, Ntub: number of spermathecal tubules, F.Ch.: length of the fertilization chamber, M.T.: length of the main tubule, LAT.t: the cumulative length of lateral tubules, TOT.t: the cumulative length of all tubules. Marked correlations (*) are significant at *p *< 0.05

The lengths of all the FPSC traits measured did not differ significantly (*p * > 0.05) neither between study sites nor monthly samples. A strong correlation was found between the length of the main tubule and the length of the fertilization chamber, while the cumulative length of lateral tubules and the cumulative length of all tubules were significantly correlated with the lengths of all spermathecal structures (Table 2).

#### Cepaea vindobonensis

**Fig. 2:**
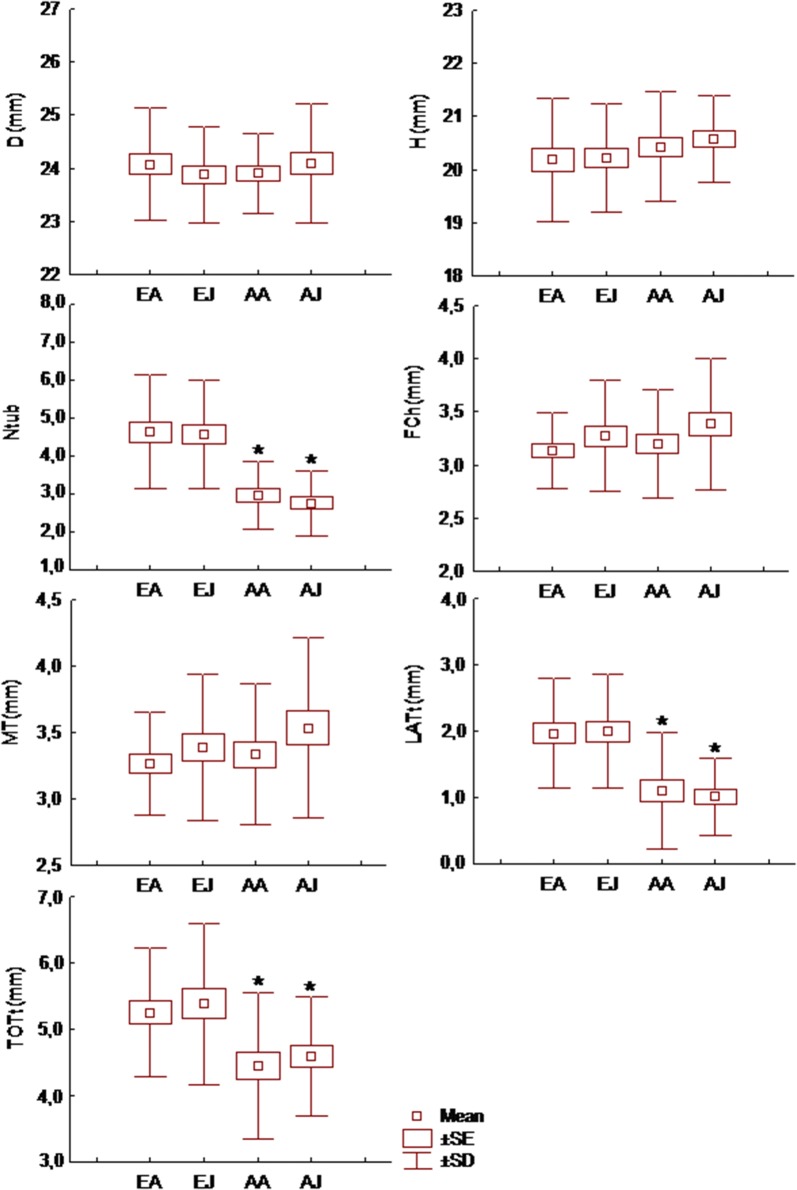
Comparison of shell size and morphological characteristics of the FPSC of *Cepaea vindobonensis* between samples taken from the two study sites. D: shell diameter, H: shell height, Ntub: number of spermathecal tubules, F.Ch.: length of the fertilization chamber, M.T.: length of the main tubule, LAT.t: the cumulative length of lateral tubules, TOT.t: the cumulative length of all tubules. EA: Edessa-April, EJ: Edessa-June, AA: Axios-April, AJ: Axios-June. Asterisks indicate significant differences at *p* < 0.05

Neither shell diameter nor shell height of snails differed significantly between the two study sites (Table 1 and Fig. 2).

The FPSC, situated in the proximal genital system, consisted of the blind-ending fertilization chamber together with the spermatheca, and parts of the hermaphrodite duct and the spermoviduct. The spermatheca consisted of several tubules. The ‘main tubule’ was only a few sections longer than the fertilization chamber in 108 out of 120 FPSC studied. Also towards its ending the ‘main tubule’ bent backwards in 41 out of 120 FPSC studied.

The number of spermathecal tubules (Ntub) ranged from 1 (only the main tubule) to 8 and a significant difference was detected between the two study sites (F = 63.165, *p *< 0.001) (Fig. 2). Snails from Edessa had 3 to 8 tubules while snails from Axios had 1 to 5 tubules in their spermathecae. No significant differences of the number of spermathecal tubules were detected between monthly samples of the same site. No significant correlation was found between the number of tubules and the size of the shell (D or H). On the contrary the number of tubules was significantly correlated with the cumulative length of lateral tubules (LATt) and the cumulative length of all tubules (TOTt) (Table 3).

**Table 3:** Coefficients of correlation between shell size traits and spermathecal traits of *Cepaea vindobonensis*

	D	H	Ntub	F.Ch	M.T.	LAT.t	TOT.t
D	1.00						
H	0.75*	1.00					
Ntub	0.17	0.09	1.00				
F.Ch	0.17	0.10	− 0.03	1.00			
M.T.	0.18	0.10	− 0.05	0.98*	1.00		
LAT.t	0.19*	0.11	0.69*	0.11	0.10	1.00	
TOT.t	0.24*	0.13	0.56*	0.62*	0.63*	0.77*	1.00

D, shell diameter; H, shell height; Ntub, number of spermathecal tubules; F.Ch., length of the fertilization chamber; M.T., length of the main tubule; LAT.t, the cumulative length of lateral tubules; TOT.t, the cumulative length of all tubules

Marked correlations (*) are significant at *p *< 0.05

The length of the fertilization chamber and the length of the main tubule did not differ significantly either between study sites or between monthly samples of the same site. On the contrary, the cumulative length of lateral tubules (LATt) (F = 38.41, *p *< 0.001) and the cumulative length of all tubules (TOTt) (F = 16.40, *p *< 0.001) differed significantly between the two study sites (Fig. 2). A strong correlation was found between the length of the fertilization chamber and the length of the main tubule, while the cumulative length of all tubules (TOTt) was significantly correlated with the lengths of all spermathecal structures and with shell diameter D (Table 3).

## Discussion

This study describes the basic morphological pattern of the FPSC of two simultaneously hermaphroditic snail species and reports intraspecific morphological variation which correlate to sperm storing capacity of the organ in populations subjected to differential selective pressures. In both species this complex organ consists of a simple fertilization chamber and a spermatheca with several tubules. The longest tubule was considered as the main tubule from which several lateral tubules emerged. The main tubule was longer than the fertilization chamber in the vast majority of the snails examined in both species. Intraspecific morphological variation was observed in both species. In both species the populations studied differed significantly in the number of spermathecal tubules but only in *C. vindobonensis* this difference led to a significant difference in the cumulative length of the tubules which correlate to the sperm storing capacity of the organ.

The basic pattern of the FPSC reported in this study has already been described for other helicids, but, despite its consistency, interspecific differences exist in the relative length of the spermatheca vs the length of the fertilization chamber. In *A. arbustorum* [9, 24], in *H. lucorum*, and in *C. vindobonensis* [33 and present study] the length of the main tubule of the spermatheca exceeds the length of the fertilization chamber in the vast majority of the snails studied. On the contrary, in *C. aspersum* [10] and in *Helix aperta* [27] the fertilization chamber appears to be the longest structure exceeding the length of the spermatheca. The structural pattern of the FPSC appears to be consistent in different populations of the species studied, characterized by different density [9, 27] or microclimatic conditions and mating propensity [10], therefore appearing unaffected by environmental factors or selective pressures.

Despite the consistency of the pattern the number and the lengths of the spermatheca tubules which correlate to the sperm storage capacity of the organ vary greatly in the snail species studied. The number of tubules in the spermatheca of *H. lucorum* (5–16 tubules) was similar to the number reported for *C. aspersum* (4–19 tubules [10]; 3–13 tubules [35]) but it was greater than that of *A. arbustorum* (2–9 tubules [9]; 1–5 tubules [36]; 2–7 tubules [22, 25]), *Helix pomatia* (3–5 tubules [37]), *C. vindobonensis* (1–8 tubules [33 and present study]) and *Helix aperta* (3–9 tubules [27]). Furthermore, the number of spermathecal tubules varied considerably among snails within populations and also between populations. In both species snails from Edessa had more tubules in their spermathecae than snails from Axios. Since no difference was detected in the lengths of spermathecae in both species (as deducted by main tubule and fertilization chamber length) the significant difference in the cumulative length of all tubules, which indicated greater sperm storage capacity, was a result of the greater number of tubules in the spermatheca of *C. vindobonensis.* In *H. lucorum* though, the difference in tubules number did not result in significant difference of cumulative tubules length and probably sperm storage capacity.

Sperm storage capacity of the spermathecae has been considered to reflect sperm competition level, the latter being approximated in several studies by population density, or environmental characteristics favouring more frequent copulations or prolonged reproductive activity [27, 29].

For *H. lucorum* the two study sites differed both in density and habitat characteristics. The population from Edessa had greater density and also extended reproductive period relative to the population from Axios [31], which presumably allowed for more mating opportunities and hence sperm competition level. Furthermore, a study on the dart and mucus gland morphometry of the same populations revealed that snails from the population of Edessa had more and longer mucus glands resulting in more mucus production [38]. The greater number of spermathecal tubules found in the Edessa population could be interpreted as showing a tendency towards a greater spermathecal complexity despite the fact that it did not lead to significantly greater cumulative tubules length that would indicate an increased sperm storage capacity.

For *C. vindobonensis* the two study sites differed only in population density. Population density in Axios was greater than in Edessa but the duration of the reproductive period was the same in both sites. Contrary to what would be expected, the snails from the less dense population in Edessa had spermathecae with greater number of tubules which resulted in significantly greater cumulative tubules length indicating increased sperm storage capacity.

The few studies that exist until now for land snails have shown contradictory results regarding the relation of spermatheca complexity to sperm competition levels. While Baminger and Haase [9], did not reveal any relationship between the degree of complexity of the spermatheca of *Arianta arbustorum* and sperm competition intensity, Abdelli et al. [27], revealed a strong correlation of spermatheca storage capacity to population density in *Helix aperta*. In both studies population density was used as a proxy for sperm competition level. One possible explanation for the contradictory results could be that sperm competition intensity does not relate to spermatheca complexity, as suggested by an empirical study on six populations of *C. aspersum* [10]. Furthermore, factors other than population density, such as habitat humidity and precipitation regimes, which influence snails’ activity and thus the number of copulations achieved, may exert a stronger influence on sperm competition level. For instance, *C. aspersum* populations studied in habitats of contrasting humidity regimes in Greece showed differences in several traits of their genital morphology, such as spermathecal tubules, number and length [29] or dart length, mucus glands number and length [38] which were related not only to population density but also to habitat characteristics.

According to the results of this study, population density does not seem to influence sperm storage capacity of both species as this trait does not differ significantly in *H. lucorum* populations and more importantly the significant increase of spermatheca complexity observed in *C. vindobonensis* is at the opposite of the anticipated direction. Furthermore, the two species may respond in a different way to sexual selection pressures posed on them as has been reported for crickets species [39]. For *H. lucorum* selective forces may influence other parts of the reproductive system such as the dart apparatus [38] while for *C. vindobonensis* habitat humidity regime may be more important in determining sperm competition level.

The absence of any correlation between shell diameter and spermatheca length (main tubule length, fertilization chamber length) or number of spermathecal tubules, indicates that the fertilization pouch-spermatheca complex (FPSC) is developmentally independent from body size as already reported for most other snail species studied like *A. arbustorum* [9, 24, 25, 36], *Cornu aspersum* [10, 29] and *Helix aperta* [27]. On the other hand, Beese et al. [22], found a significant positive correlation between snail size and spermatheca length as well as spermatheca volume. In this study, the only significant correlation detected was the correlation between shell diameter and cumulative tubule length in *C. vindobonensis* snails. Furthermore, the cumulative tubule length was significantly correlated to the length of all other spermathecal structures and also to the number of the spermathecal tubules in both species, as has also been reported for *A. arbustorum* by Baminger and Haase [9].

## Conclusion

Overall our results indicate that the two investigated snail species respond in a different way to selective pressures such as population density and habitat microclimate, which may be indicative for the level of sperm competition, regarding the structure of the fertilization pouch-spermatheca complex and thus their sperm storage capacity. It is well known from studies on other animal taxa [40] that variation in ecological conditions often drives the evolution of adaptive mating strategies leading to diversification of mating systems and variability of sexual selection pressures. This variability may be seen as a side effect of variation in factors affecting copulation opportunities such as population density or habitat specificity [22]. Population density has long been investigated as a factor affecting sperm competition level, but only recently researchers have started to consider environmental conditions as equally important in affecting sexual selection form and direction [41–43]. Very few species of land snails have been studied under this framework until now and the results obtained are largely contradictory hindering the formation of a general pattern. Clearly, an intensification of research in more species would add information needed to further clarify this issue.
